# Yeast Genetic Analysis Reveals the Involvement of Chromatin Reassembly Factors in Repressing HIV-1 Basal Transcription

**DOI:** 10.1371/journal.pgen.1000339

**Published:** 2009-01-16

**Authors:** Manuela Vanti, Edurne Gallastegui, Iñaki Respaldiza, Alfonso Rodríguez-Gil, Fernando Gómez-Herreros, Silvia Jimeno-González, Albert Jordan, Sebastián Chávez

**Affiliations:** 1 Departamento de Genética, Universidad de Sevilla, Seville, Spain; 2 Centre de Regulació Genòmica, Universitat Pompeu Fabra, Barcelona, Spain; Stanford University School of Medicine, United States of America

## Abstract

Rebound of HIV viremia after interruption of anti-retroviral therapy is due to the small population of CD4+ T cells that remain latently infected. HIV-1 transcription is the main process controlling post-integration latency. Regulation of HIV-1 transcription takes place at both initiation and elongation levels. Pausing of RNA polymerase II at the 5′ end of HIV-1 transcribed region (5′HIV-TR), which is immediately downstream of the transcription start site, plays an important role in the regulation of viral expression. The activation of HIV-1 transcription correlates with the rearrangement of a positioned nucleosome located at this region. These two facts suggest that the 5′HIV-TR contributes to inhibit basal transcription of those HIV-1 proviruses that remain latently inactive. However, little is known about the cell elements mediating the repressive role of the 5′HIV-TR. We performed a genetic analysis of this phenomenon in *Saccharomyces cerevisiae* after reconstructing a minimal HIV-1 transcriptional system in this yeast. Unexpectedly, we found that the critical role played by the 5′HIV-TR in maintaining low levels of basal transcription in yeast is mediated by FACT, Spt6, and Chd1, proteins so far associated with chromatin assembly and disassembly during ongoing transcription. We confirmed that this group of factors plays a role in HIV-1 postintegration latency in human cells by depleting the corresponding human orthologs with shRNAs, both in HIV latently infected cell populations and in particular single-integration clones, including a latent clone with a provirus integrated in a highly transcribed gene. Our results indicate that chromatin reassembly factors participate in the establishment of the equilibrium between activation and repression of HIV-1 when it integrates into the human genome, and they open the possibility of considering these factors as therapeutic targets of HIV-1 latency.

## Author Summary

Acquired immunodeficiency syndrome (AIDS) is caused by the human immunodeficiency virus (HIV). Drugs used for anti-viral therapy are very efficient in controlling the presence of viral particles in infected patients. However, if this therapy is interrupted, rebound of viremia occurs due to the small population of cells that remain latently infected. A specific region of the HIV genome (the beginning of the transcribed region) plays an important role in viral expression and may contribute to its silent state in latently infected cells. We reconstructed a minimal HIV system in yeast to perform a genetic analysis of the role played by that specific region of the viral genome. We found that the repressive role played by this region is mediated by a group of proteins (chromatin reassembly factors) so far associated with other functions during gene expression. We confirmed that this group of factors plays a role in controlling HIV-1 basal transcription in human cells.

## Introduction

Following integration into the host cell genome, HIV-1 transcription is the most important step regulating viral replication. The main factor involved in this regulation is the viral Tat protein, which binds TAR, a structured RNA element present at the 5′ end of the viral mRNAs. The structure of the mRNA 5′ end also contributes to the pausing of RNA polymerase II (RNApolII) at the LTR [1]. This pausing is characteristic of HIV-1 transcription and appears to play a role in maintaining low levels of basal transcription when the promoter is not activated [2],[3].

Tat activates transcription by both inducing chromatin remodeling and recruiting P-TEFb, a cell factor required for productive transcription elongation, onto the viral LTR [4],[5]. Tat also stimulates transcription by direct, TAR-independent activation of the viral promoter [6]. Induction of the host transcription factors NF-α B cooperates with Tat in completing HIV activation [7].

Chromatin plays an essential role in the transcriptional regulation of HIV (reviewed by [8],[9]). The transition from basal to activated transcription correlates with drastic changes in the acetylation levels of the nucleosomes covering the HIV promoter [10] and with the rearrangement of nucleosome positioning on the 5′ LTR [11]. These chromatin alterations are catalyzed by histone modifying enzymes and ATP-dependent chromatin remodeling complexes, which are recruited by Tat to the LTR [12]–[14]. Tat action on HIV chromatin is also mediated by the nucleosome assembly protein hNAP-1 [15].

Several host factors, including the receptor tyrosine kinase RON [16] and a subunit of the CPSF complex [17], contribute to maintaining the repressive state characteristic of HIV latency, but most elements directly responsible for HIV postintegration latency are also related to chromatin. Histone deacetylases (HDAC) are involved in the transcriptional repression of the LTR [18] and their recruitment by CBF-1 promotes HIV-1 entry into latency [19]. HP1, binding trimethylated histone H3-K9, also plays a role in HIV-1 silencing [20]. Consistent with this role of chromatin in HIV latency, the chromatin environment of the integration site influences the transcriptional behavior of the provirus, whose level of basal transcription is undetectable in some integrants [21].

One of the most interesting phenomena that takes place during the transcriptional activation of a latent HIV-1 provirus is the precise, transcription-independent remodelling of nucleosome-1, positioned at the 5′ end of HIV-1 transcribed region (5′HIV-TR), immediately downstream of the transcription start site [11]. In this work, we performed a genetic analysis of the role of the 5′HIV-TR in basal transcription, making use of the yeast *Saccharomyces cerevisiae*, which has already been successfully used to investigate other aspects of HIV-1 biology [22]–[24]. We show that the 5′HIV-TR is critical in repressing basal transcription in yeast and that this phenomenon is mediated by FACT, Spt6 and Chd1, proteins involved in co-transcriptional chromatin reassembly. Finally, we confirm that this group of factors plays a role in maintaining low levels of basal transcription in human cells.

## Results

### The 5′HIV-TR Inhibits Basal Transcription in Yeast

**Figure 1:**
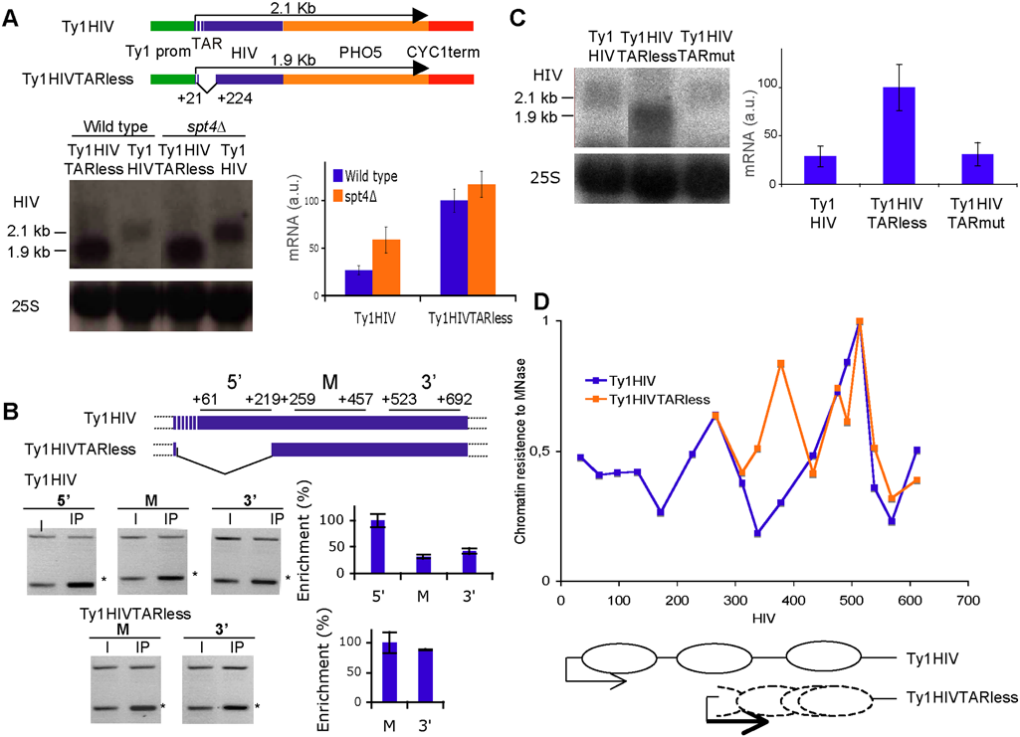
Influence of the 5′ end of HIV-1 transcribed region on basal transcription in yeast. (A) The 5′HIV-TR inhibits Ty1-HIV-1 expression and this inhibition partially depends on yDSIF. mRNA samples from the BY4741 wild-type yeast strains and from an isogenic *spt4Δ* strain, transformed with plasmids pTy1-HIV and pTy1-HIVTARless, were resolved in agarose gels and analyzed by Northern blotting. Quantification of the signals is shown, after normalizing with the levels of 25S rRNA. A typical result and the quantification of three independent experiments are shown. (B) ChIP analysis of RNApol II in the two indicated transcription units. ARG3 cells, isogenic to BY4741, except for the *RPB1::cMyc* allele, transformed with pTy1-HIV and pTy1-HIVTARless, were grown to mid-log phase. Cross-linked chromatin was immunoprecipitated with monoclonal anti-Myc antibody. PCR was conducted on two dilutions of whole cell extract (WCE) and two different amounts of immunoprecipitated DNA (IP; only the most diluted are shown). PCR primers flank segments located in the 5′, central and 3′ regions of each gene. Diagrams at the top indicate the position of the PCR amplicons relative to the HIV-1 transcription start site. A non-transcribed region adjacent to *FUS1* was used as a control. A typical experiment for each gene is shown on the left and the averages of three independent experiments are quantified on the right. The HIV signals are marked with an asterisk. (C) The TAR RNA structure is not required for the repressive effect of the 5′HIV-TR on basal transcription. Northern analyses of Ty1-HIV, Ty1-HIVTARless and Ty1HIVTARmut in BY4741 were performed as in A. (D) Chromatin configuration of the 5′HIV-TR in yeast. Spheroplasts of formaldehyde-treated BY4741 cells containing pTy1-HIV and pTy1-HIVTARless were lysed and digested with MNase. After purification, DNA was quantified with real-time PCR as described in Materials and Methods, using the primers listed in Table S3. Signals were normalized against naked DNA by repeating the same procedure with control DNA, purified from formaldehyde-treated cells before digesting it with MNase. For comparison, the two profiles were represented as fractions of the signal obtained with amplicon 14 (+490/+537). Diagrams represent the estimated locations of positioned nucleosomes. Dashed ovals indicate possible alternative locations of nucleosomes in TyHIVTARless.

It has been previously described how the entire HIV-1 LTR is transcriptionally inactive in yeast [25]. Therefore, in order to investigate transcription elongation through the 5′HIV-TR in *Saccharomyces cerevisiae*, we constructed a chimeric yeast-HIV transcription unit. We located a fragment of the HIV-1 transcribed region (+1/+671), under the transcriptional control of a Ty1 promoter, which drives a retroelement with a low, but detectable, level of basal transcription [26]. The fragment included all the sequences that have been shown to be relevant in regulating HIV-1 transcriptional elongation. We did not choose a longer piece of HIV-1 to avoid the complex pattern of spliced forms that characterize this virus. In order to ensure its detection by northern blot, we increased the length of the mRNA by adding the coding region of the yeast *PHO5* gene (Figure 1A). A transcript of the expected length (2.1 kb) was detected when we transformed three different wild-type strains of *Saccharomyces cerevisiae* with a centromeric plasmid containing the Ty1-HIV construct (Figures 1A and Figure S1).

In order to explore whether the 5′HIV-TR can influence transcription in yeast, we deleted a 203 bp fragment of this region, including most of the TAR-encoding R domain. The new transcription unit, Ty1-HIVTARless, also expressed a transcript of the expected length (1.9 kb). Quantification of the transcripts revealed that the deletion produced a clear increase in the mRNA amounts (Figures 1A and Figure S1). To confirm that this difference was due to RNApolII pausing, we performed ChIP experiments with a Myc-tagged form of Rpb1, the biggest subunit of RNApolII. The results obtained with Ty1-HIV showed that the amounts of RNApolII bound to the 5′HIV-TR were higher than the levels detected downstream (Figure 1B). This enrichment was not due to the proximity to the initiation site, since we did not detect a significant accumulation of RNApolII at the equivalent region of Ty1-HIVTARless, immediately downstream of its initiation site (Figure 1B).

One of the main factors regulating HIV-1 transcription is DSIF, which exerts a negative influence on RNApolII transcription during early elongation [27]. We analyzed the mRNA levels of Ty1-HIV and Ty1-HIVTARless in an *spt4Δ* mutant lacking one of the subunits of yeast DSIF. As expected, the absence of Spt4 partially abolished the repressive role of the 5′HIV-TR (Figure 1A).

In order to ascertain whether the TAR structure contributes to the repressive role of the 5′HIV-TR in yeast, we constructed a mutant version of Ty1-HIV (Ty1-HIVTARmut), in which we replaced the promoter-distal part of the TAR-encoding sequence (5′-GCTCTCTGGCTAACTAGGGAACCC-3′) by a complementary string (5′-CGAGAGACCGATTGATCCCTTGGG-3′). The new transcription unit, encoding an mRNA without the ability to form a TAR structure, showed levels of expression similar to Ty1-HIV and clearly below Ty1-HIVTARless (Figure 1C), indicating that the phenomenon that we are describing does not depend on the TAR element.

Non-active HIV-1 LTR is occupied by a set of positioned nucleosomes, one of which covers the 5′HIV-TR [11]. To complete the characterization of Ty1-HIV, we digested chromatin and naked DNA samples with micrococcal nuclease (MNase) and analyzed nuclesome positioning by quantitative PCR. As shown in Figure 1D, the transcribed region of Ty1-HIV showed a clearly defined nucleosomal pattern, similar to HIV-1 in human cells. In contrast, the pattern on the Ty1-HIVTARless transcribed region is incompatible with a unique translational phase of nucleosomes, suggesting a more dynamic chromatin structure with several alternative distributions (Figure 1D). The higher accessibility of Ty1-HIVTARless chromatin was also confirmed by hybridization of MNase–treated samples with probes covering the 5′end of its transcribed region (Figure S2). Hybridization of Ty1-HIV samples with probe 1, covering the 5′HIV-TR produced a ladder of signals, compatible with a regular nucleosomal structure, whose shortest fragment was approximately 150 bp long. Similar results were obtained with probe 3. In contrast, hybridization of Ty1-HIVTARless samples with probe 2, covering the 5′ end of its transcribed region, produced a nucleosomal ladder that included a smeared signal of DNA fragments shorter than 100 bp. Rehybridization with probe 3, also showing a less regular nucleosomal ladder, excluded the possibility that this smear was due to DNA degradation (Figure S2). Taken together, the results shown so far indicate that Ty1-HIV is a good tool for investigating transcription elongation through the 5′HIV-TR, and suggest that the repressive role of this DNA element in yeast is related to its chromatin structure.

**Figure 2:**
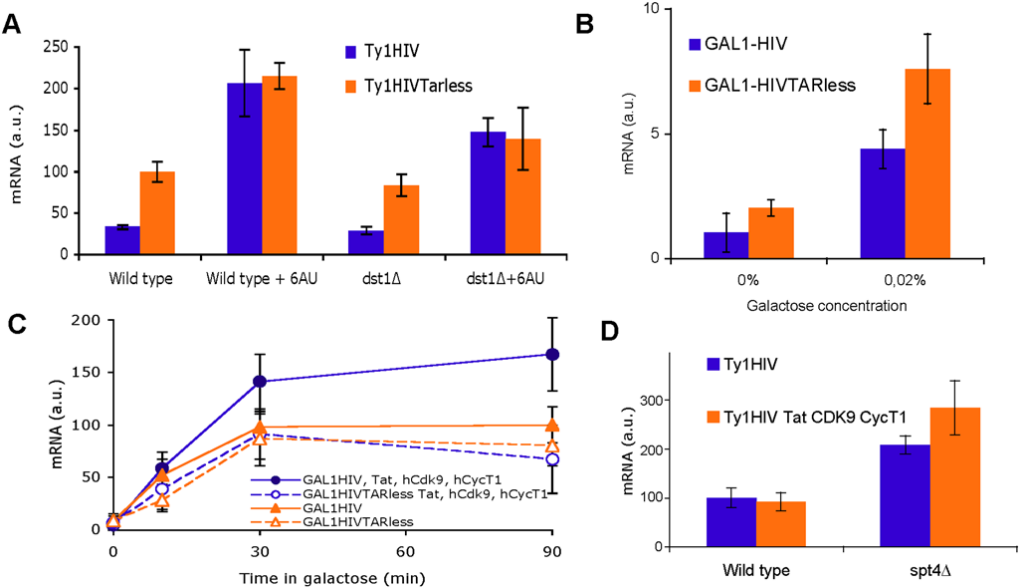
No effect of the 5′HIV-TR on mRNA levels upon promoter activation. (A) Activation of the Ty1 promoter by the NTP-depleting agent 6-azauracil eliminates the inhibitory effect of the 5′HIV-TR, both in a wild type and in a strain lacking TFIIS (*dst1Δ)*. Experiment details as for Figure 1A. (B) The 5′HIV-TR inhibits *GAL1*-driven transcription when the promoter is weakly active. Cells transformed with plasmids pGAL1-HIV or pGAL1-HIVTARless were grown to mid-log phase in minimal medium with raffinose (0%) or with raffinose plus 0.02% galactose (0.02%) as carbon sources. (C) No effect of the 5′HIV-TR on mRNA levels is detected when an activated GAL1 promoter drives transcription, unless Tat and P-TEFb are present in the yeast cell. Cells transformed with plasmids pGAL1-HIV or pGAL1-HIVTARless, alone or together with p415GPD-CycT1, p414GPD-Cdk9 and p413GPD-Tat, were grown to mid-log phase as in B and incubated for 90 min in the presence of 2% galactose. (D) Tat and P-TEFb enhance Ty1-HIV expression in *spt4Δ* but they do not alter basal transcription in the wild type. Cells transformed with plasmids pTy1-HIV or pTy1-HIVTARless, alone or together with p415GPD-CycT1, p414GPD-Cdk9 and p413GPD-Tat, were grown to mid-log phase. In all cases, mRNA samples were taken and analyzed by Northern blot as in Figure 1A and the averages of three independent experiments are shown.

It has been described how nucleotide deprivation stimulates Ty1 transcription [28]. We found that the addition of 6-azauracil, an NTP-depleting drug, to the medium produced a rapid increase in the overall level of Ty1 mRNA (Figure S3). We made use of this simple method of activating the Ty1 promoter in order to study the effect of promoter activation on 5′HIV-TR transcription. We observed that promoter activation eliminated the functional differences between Ty1-HIV and Ty1-HIVTARless, which became equally expressed in the presence of 6-azauracil. Similar results were obtained in the absence of the RNApolII cleavage-factor TFIIS (*dst1Δ*), suggesting that RNApolII does not become arrested when transcribing the 5′HIV-TR (Figure 2A).

To further confirm that promoter activation abolishes the repressive effect of the 5′HIV-TR, we replaced the Ty1 promoter by the one of *GAL1*, a tightly regulated gene that becomes strongly activated when galactose is the carbon source. Under conditions of weak activation (2% raffinose plus 0.02% galactose) a clear difference was observed between GAL1-HIV and GAL1-HIVTARless (Figure 2B), indicating that the 5′HIV-TR does not only repress Ty1-driven transcription. As expected, when we added high levels of galactose (2%) to the medium, we observed a further increase in mRNA accumulation. However, in this case, the levels of mRNA accumulation were the same in cells containing the GAL1-HIV construct as in those transformed with GAL1-HIVTARless (Figure 2C). This result shows again that the repressive role of the 5′HIV-TR is not effective under activating conditions.

When HIV-1 promoter becomes active in human cells, the TAR domain is required for full activation by Tat and P-TEFb. We wondered whether the activation of the promoter in yeast still allows a supplemental induction by Tat and P-TEFb. We expressed hCDK9, hCycT1 and Tat by cloning their cDNAs in yeast expression vectors (Figure S4A and B). We did not detect any influence of Tat and P-TEFb on Ty1-HIV basal transcription (Figure 2D). We were also interested in testing the effect of Tat and P-TEFb on Ty1-HIV expression under activating conditions. The addition of 6AU to these cells produced very irregular results, likely due the presence of three different plasmids in this strain and to the plasmid instability produced by 6-azauracil [29]. As an alternative approach, we tested the effect of Tat and P-TEFb on a more active Ty1-HIV by repeating the assay in an *spt4Δ* strain. In this case we obtained consistent results and a weak but significant effect was detected (Figure 2D). We also detected a positive effect of P-TEFb on activated GAL1-HIV expression, which was strictly dependent on CycT1 and partially dependent on CDK9 (Figure S4C). We analyzed whether this second level of activation was mediated by the 5′HIV-TR, encoding the TAR RNA domain. As expected, Tat and P-TEFb were unable to enhance the expression of GAL1-HIVTARless (Figure 2C). This set of results suggests that the repressive role of the 5′HIV-TR and the TAR-dependent regulation of HIV-1 transcription can be functionally separated.

### Genetic Analysis of the 5′HIV-TR Repressive Function

We then analyzed the expression of Ty1-HIV and Ty1-HIVTARless in a selected group of mutants related to the elongation step of transcription. The results of these analyses are shown in Figure S5 and 3A. Some of the mutants tested, such as *swi2Δ*, lacking a subunit of the SWI-SNF chromatin remodeling complex, *set1Δ*, affecting the COMPASS histone-methylation complex, or *rpd3Δ*, lacking one of the main histone deacetylases, did not produce a significant effect on the expression of any of the two chimeric transcription units. We concluded that these factors do not contribute significantly to either elongation through the 5′HIV-TR or Ty1 promoter activity.

**Figure 3:**
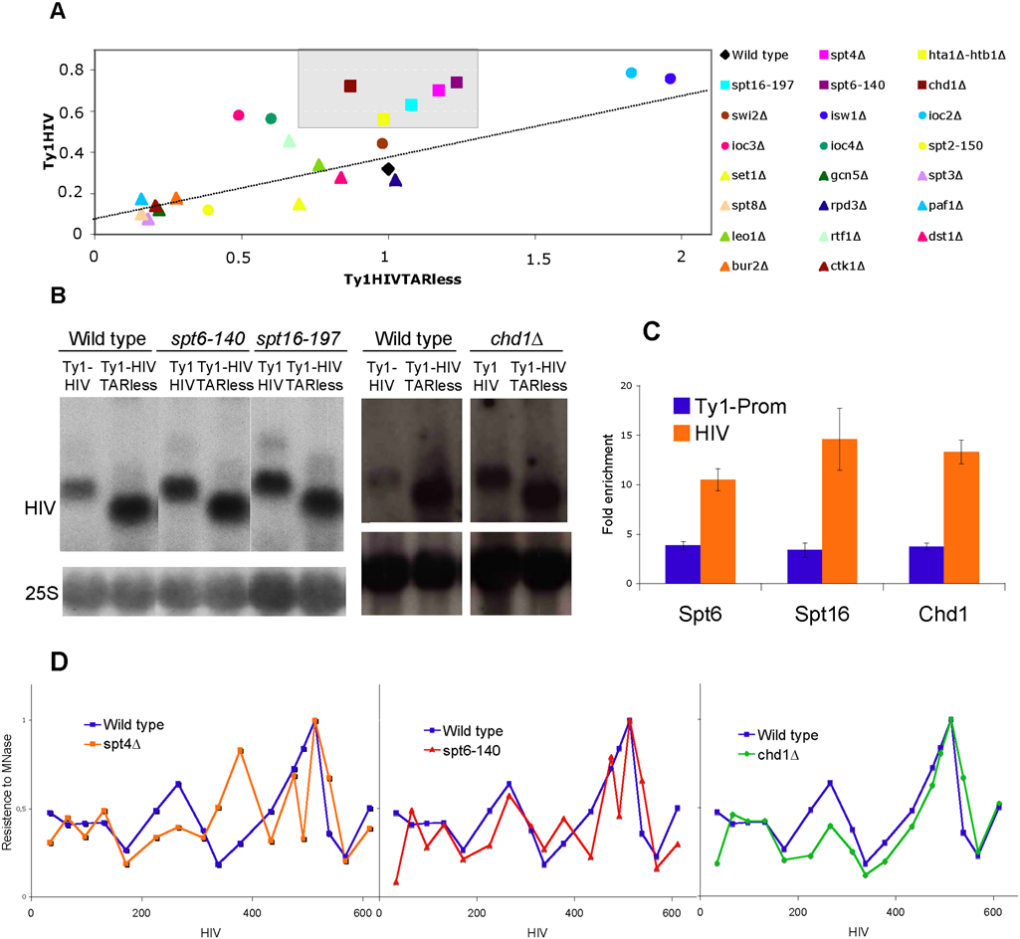
Genetic analysis of the 5′HIV-TR inhibitory effect. (A) The inhibitory role of the 5′HIV-TR is compromised in mutants affecting co-transcriptional chromatin reassembly. mRNA samples of the indicated mutants, transformed with pTy1-HIV and pTy1-HIVTARless, were analyzed by Northern blot, as in Figure 1A. Averages of three independent experiments (see Figure S5) were plotted. Dashed line represents all possible variations of mRNA levels that maintain the Ty1HIV/Ty1HIVTARless proportion of the wild type. The grey square covers all points with a level of Ty1HIV mRNA significantly higher than the wild type (more than two standard deviations), but with a similar level of Ty1HIVTARless mRNA that the wild type (within two standard deviations). (B) Representative Northern blots, comparing the mRNA levels of Ty1-HIV and Ty1-HIVTARless, in *spt6-*140 (FY137), *spt16-197* (FY348), *chd1Δ* and their respective isogenic wild-type strains (FY120 and BY4741). (C) ChIP analysis of Spt6, Spt16 and Chd1. SJY25, DBY871 and DBY969 strains, transformed with pTy1-HIV, were grown to mid-log phase. Cross-linked chromatin was immunoprecipitated and quantified by real-time PCR as described in Materials and Methods. Averaged fold enrichments of three independent experiments, relative to an untranscribed telomeric region, are shown. (D) Chromatin configuration of the 5′HIV-TR in *spt4Δ*, *spt6-140* and *chd1Δ*. Averages of three independent experiments are shown. Experimental details as in Figure 1D.

Some other mutants, such as *paf1Δ*, lacking the main subunit of the PAF1 complex; *spt2-150*, affected in a HMG component of yeast chromatin; those affecting the SAGA complex (*gcn5Δ*, *spt3Δ*, *spt8Δ*), or those lacking elements of cyclin-dependent kinases involved in transcription (*bur2Δ*, *ctk1Δ*) produced a negative effect on the expression of both Ty1-HIV and Ty1-HIVTARless (Figure S5). The simplest interpretation of these results is that this set of factors is required for the basal activity of the Ty1 promoter and/or for transcription elongation through both Ty1-HIV and Ty1-HIVTARless. In any case, it seems that these factors are not related to the repressive effect of the 5′HIV-TR.

The absence of Isw1, the catalytic subunit of the ISW1 chromatin remodeling complexes, stimulated the expression of both Ty1-HIV and Ty1-HIVTARless (Figure S5). This result is fully consistent with the described role of Isw1 in inhibiting Ty1 expression [30]. A similar result was obtained with the *ioc2Δ* mutant, lacking one of the subunits of the ISW1b complex. However, the absence of Ioc3 or Ioc4, belonging to the ISW1a and ISW1b complexes respectively, enhanced the expression of Ty1-HIV but decreased that of Ty1-HIVTARless (Figure S5). These results confirm the intricacy of the roles played by the ISWI complexes [31] and might suggest their involvement in the repressive effects of the 5′HIV-TR, but are difficult to interpret properly without further research.

A last group of mutants included *chd1Δ*, lacking a chromodomain protein involved in transcription elongation; *spt16-197*, affecting one of the subunits of the FACT elongation factor; and *spt6-140*, encoding a defective form of a factor related to chromatin and mRNA transactions during elongation [32]–[34]. Both *SPT16* and *SPT6* are essential genes and, like *CHD1*, are involved in the reassembly of chromatin during transcription elongation [35]. Strong functional interactions have been detected amongst these three genes [36]–[38]. This group of mutants did not significantly affect the expression of Ty1-HIVTARless but did increase the levels of Ty1-HIV mRNA, a phenotype very similar to that of *spt4Δ* (Figure 3A, 3B and S5).

Spt16, FACT and Chd1 are general chromatin factors that are associated with actively transcribed regions in all eukaryotes investigated so far [39]–[41]. Since they seemed to play a role in the weakly transcribed Ty1-HIV, we tested the presence of these factors on this transcription unit by ChIP. We found significant enrichments for the three proteins on Ty1-HIV (Figure 3C). In all three cases, the enrichment was higher on the transcribed region than on the promoter, confirming the well-known connection of these factors with transcription elongation.

These results suggest that the repressive function of the 5′HIV-TR depends on chromatin reassembly. To further corroborate this hypothesis, we analyzed the expression of Ty1-HIV and Ty1-HIVTARless in *hta1Δ-htb1Δ*, a mutant suffering from a deficit of H2A and H2B histones. The expression of Ty1-HIVTARless was not significantly affected in this mutant, whereas the mRNA levels of Ty1-HIV showed a clear increase (Figure 5S). Thus, *hta1Δ-htb1Δ* grouped with *spt4Δ* and the three chromatin-reassembly mutants (Figure 3A).

In order to verify that this group of mutants was indeed affecting the chromatin structure of Ty1-HIV, we analyzed nucleosome positioning on Ty1-HIV in some of the mutants (Figure 3D). *spt4Δ* caused a general alteration of nucleosome positioning on the transcribed region of Ty1-HIV, clearly affecting nucleosomes 1, 2 and 3. In the overlapping region, this pattern was very similar to the one exhibited by Ty1-HIVTARless in the wild type (Figure 1D). *spt6-140* also showed a less positioned pattern than the wild type, although in this case nucleosome 2 was almost unaffected. In turn, *chd1Δ* almost eliminated the signal corresponding to nucleosome 2 without significantly affecting the other two nucleosomes (Figure 3D).

### Chromatin Reassembly Factors Contribute to Repressing HIV-1 Basal Transcription in Human Cells

In order to investigate whether chromatin reassembly factors really do play a role in the repression of HIV-1 basal transcription in human cells, we knocked down the expression of human Spt6 and Chd1 in HIV latently infected cells. A model of HIV-1 latency in Jurkat cells infected with an HIV minigenome encoding Tat and GFP has been previously reported [42]. Upon infection, latent cells are defined as those that do not express constitutively GFP, but need further stimuli of HIV promoter by mitogens (PMA) or cytokines (TNF-α). After removal of stimuli, the HIV promoter becomes repressed again and GFP-negative cells can be purified and maintained as a population; alternatively, individual cells representing unique latent viral integrations can be cloned. These cells are useful for investigating maintenance of HIV promoter repression and viral latency. We hypothesized that, if chromatin reassembly factors were involved in HIV promoter repression, its depletion would cause gene reactivation.

**Figure 4:**
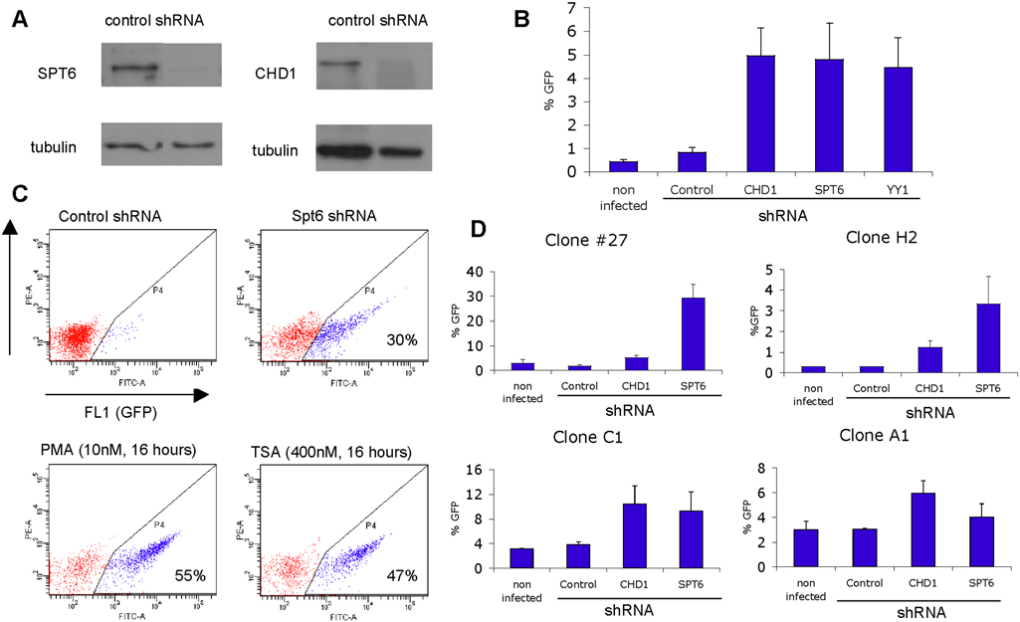
shRNA-mediated depletion of chromatin reassembly factors hSpt6 and hChd1 in Jurkat cells derepress latently-integrated HIV. (A) shRNA-mediated depletion of SPT6 and CHD1. A population of HIV (LTR-Tat-IRES-GFP-LTR) latently-infected human cells was infected with Control, Spt6 (#1, target sequence CGCCTTGTACTGTGAATTTAT) or Chd1 (#3, GCAGTTGTGATGAAACAGAAT) shRNA expression lentiviruses (pLKO.1-Puro) and, 10 days after puromycin (2 mg/ml) selection, depletion of these factors was tested in Western blot with specific antibodies and tubulin as a loading control. (B) HIV reactivation in a heterogeneous population of HIV latently infected Jurkat cells, expressed as percentage of cells that become GFP-positive 10–15 days after infection with the indicated shRNA-expressing lentiviruses and continuous selection with puromycin. Values represent the mean±SD of three independent infection experiments. (C) Example of FACS analysis of GFP expression as a result of HIV promoter reactivation in a latently infected Jurkat clone after infection with a Spt6 specific shRNA-expressing vector or treatment with PMA (10 nM for 16 h) or TSA (400 nM for 16 h). The percentage of GFP-positive cells is indicated inside the corresponding gate. (D) HIV reactivation in individual cell clones. Four different clones containing single HIV latent integrations previously mapped were infected with the indicated shRNA-expressing lentiviruses, selected by puromycin treatment, and GFP expression was monitored by FACS over time. Values represent the mean±SD of percentage of GFP-positive cells of two independent experiments 10–15 days after infection.

We used lentiviral shRNA expression vectors to deplete Spt6 and Chd1 in HIV latently infected cell populations (Figure 4A). 10–15 days after shRNA infection and puromycin selection, the percentage of Spt6 and Chd1 knocked-down cells expressing GFP reached ca. 5%, a significantly higher proportion that obtained with a control shRNA vector (Figure 4B). Because the latently-infected cell population is heterogeneous, representing a myriad of HIV integrations at different genome locations and chromatin environments, only a small proportion of latent HIV provirus are activated by solely depleting these factors. A similar degree of reactivation was obtained with an shRNA against YY1, a host transcription factor previously reported to be involved in histone deacetylase (HDAC) recruitment and HIV promoter repression [43],[44] (Figure 4B). The HDAC inhibitor trichostatin A (TSA) activates the HIV promoter in ca. 9.5% of the latently-infected cell population, indicating again that not all HIV integrations are equally sensitive to the inhibition or depletion of a repressive chromatin factor (data not shown).

Next, we investigated the effect of Spt6 and Chd1 depletion on HIV expression in particular clones containing single latent integrations either in centromeric alphoid repeats (clones H2 and C1), an intergenic region (clone A1) or in an intron of the highly transcribed gene UBXD8 (clone 27) [42] (and unpublished results). The percentage of reactivated cells varied depending upon the clones, reaching ca. 30% reactivation in clone 27 after knocking-down Spt6 (Figure 4C and 4D). Reactivation by TSA is also dissimilar between clones, ranging from 27% in clone C1 to 47% in clone 27 (Figure 4C and data not shown). Similarly, Spt6 and Chd1 depletion promoted reactivation of the silent HIV promoter in a newly-generated model of HIV latency in HeLa cells (Figure S6). Moreover, these effects were observed with several different shRNA sequences and correlated well with the degree of target protein depletion achieved, discarding unspecific off-target effects (Figure S6). Altogether, our data indicates that these two chromatin reassembly factors identified in the yeast screening contribute to maintain HIV repression in infected human cells.

## Discussion

In this work we have made use of yeast genetic analysis to investigate the influence of the 5′HIV-TR on basal transcription. Several attempts to study HIV-1 transcription in yeast have been described, all of them focused in the transactivation capacity of Tat. Although a fusion of Tat with the DNA binding domain of Gal4 can activate the *GAL1* promoter [45], no Tat-dependent transactivation of the HIV-1 LTR had yet been achieved [25]. Therefore, the present work is the first successful reconstruction in yeast of a transcriptional system based on HIV-1 elements.

The artificial character of the Ty1-HIV transcription unit raises the possibility of the conclusions extracted from this work being of no relevance for HIV-1 biology. Several results presented in this piece of research argue against that point of view. We show that the 5′HIV-TR not only represses transcription driven by the Ty1 promoter but can also act on a completely different promoter (*GAL1*) when this is weakly active. We also show that the 5′HIV-TR induces an accumulation of RNApol II immediately downstream of the promoter, a very common situation throughout the human genome but extremely infrequent in yeast [46]. It has been recently proposed that the reason for this difference is the chromatin organization of the transcription start site, which is usually covered by a positioned nucleosome in yeast and immediately upstream of a positioned nucleosome in most metazoan genes [47]. We show in this work that the chromatin organization of the HIV fragment present in Ty1-HIV closely resembles the distribution of positioned nucleosomes of HIV-1 proviruses in the human genome. We also present evidence that transcription through the 5′HIV-TR in yeast is influenced by factors that have been previously shown to govern HIV-1 transcription elongation: yDSIF contributes to the repressive role of the 5′HIV-TR in basal transcription, whereas Tat and P-TEFb enhance active transcription in a 5′HIV-TR-dependent manner. Finally, the main conclusion of the genetic analysis, which is the involvement of chromatin reassembly factors in repressing HIV-1 basal transcription, has been confirmed in a human model of HIV-1 latency. Based on these considerations, we believe that the chimeric yeast-HIV system is a valid complementary tool for HIV research.

The role of the 5′HIV-TR in basal transcription has scarcely been studied, and the data available is sometimes contradictory [48],[49], likely due to the use of transiently transfected DNA, which does not ensure a proper organization of DNA in chromatin [50]. In fact, mutation in this region produced different effects on HIV-1 transcription when an integrated version was compared to a transiently transfected one [51],[52].

**Figure 5:**
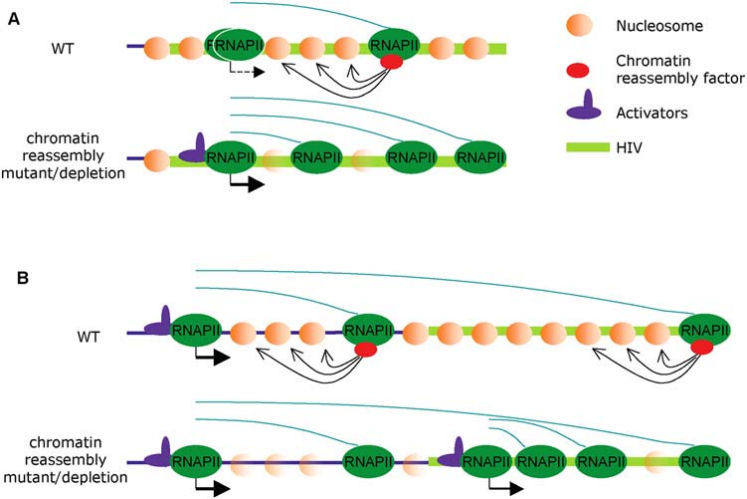
A model for the contribution of chromatin reassembly factors to the repression of HIV basal transcription. (A) Chromatin reassembly factors, recruited by the elongating form of RNApol II, stabilize nucleosomes on the transcribed region, keeping basal transcription at low rates. In the absence of chromatin reassembly factors, nucleosomal configuration of the transcribe region becomes instable, increasing basal transcription and eventually favoring promoter activation. (B) Chromatin reassembly factors may also contribute to the silencing of HIV by transcriptional interference. HIV proviruses integrated in highly expressed genes would remain untranscribed due to the repressive chromatin configuration established by chromatin reassembly factors during transcription elongation. In the absence of chromatin reassembly factors, nucleosomes repressing the 5′-LTR become instable, allowing transcription factors to activate the HIV promoter.

The data shown in the present work indicates that the 5′HIV-TR contributes to maintaining low levels of basal transcription without interfering with promoter activation. The genetic analysis that we have performed shows a contribution of chromatin reassembly factors to this repressive function of the 5′HIV-TR. We have also confirmed that Spt6 and Chd1 favor a close chromatin configuration on the transcribed region of Ty1-HIV. The role of chromatin reassembly factors at this level seems to be as significant as the one played by DSIF, since the combination of the chromatin alterations produced by *spt6-140* and *chd1Δ* fully matches those caused by *spt4Δ*. Deletion of the 5′HIV-TR causes a disruption of nucleosome positioning on the rest of the HIV fragment present in Ty1-HIVTARless, mimicking the patterns of *spt4Δ* and, to a lesser extent, of *spt6-140*. It is possible that this chromatin difference between the two transcription units is a consequence of the higher transcription level of Ty1-HIVTARless. Alternatively, the absence of the +1 nucleosome may destabilize the overall chromatin configuration and this would, in turn, give rise to increased transcriptional activity. The differences in the chromatin patterns of *spt4Δ*, *spt6-140* and *chd1Δ*, all showing similar levels of expression, indicate that the chromatin differences are likely to be responsible for the transcription increase and not vice versa. This explanation also fits better with the accumulation of RNApolII on the 5′HIV-TR and with the results of our genetic analysis. In this scenario, productive elongation would be infrequent under non-activating conditions due to both the low number of initiation events and the positioned nucleosomes sitting on the 5′HIV-TR but, in those rare occasions when RNApol II gets through the chromatin boundary, the immediate action of chromatin reassembly factors (recruited by elongating RNApolII itself) would contribute to rebuilding the repressive chromatin configuration, avoiding a transition into an activation-prone chromatin environment (Figure 5A).

Several chromatin-mediated mechanisms contribute to regulating HIV-1 transcription [8]: the activation of the LTR promoter is mediated by the acetylation state of its chromatin, especially by the nucleosome located upstream in the LTR (nucleosome 0) [10]. An additional role of chromatin in regulating HIV-1 transcription takes place at the level of early elongation, since the positioned nucleosome covering the 5′HIV-TR (nucleosome 1) becomes remodeled in response to promoter activation, in a transcription-independent manner [11].

Although transcription of Ty1-HIV is more intensively repressed by chromatin reassembly factors than Ty1-HIVTARless, we do not believe that the 5′HIV-TR is specifically required for their recruitment. Spt6, FACT and Chd1 are general elongation factors, whose association to actively transcribed regions, in an RNA polymerase II-dependent manner, is well documented from yeast to metazoa [33], [38]–[41], [53]–[55]. We favour the idea of the 5′HIV-TR being an optimal DNA sequence for nucleosome positioning. Recent genome-wide studies show the importance of 5′ sequences in specifying the location of +1 nucleosomes. In turn, these act as barriers against which other nucleosomes are packed [56]. In such a DNA context the repressive action of chromatin reassembly factors would be maximal.

Basal transcription of HIV-1 is highly dependent on the chromatin environment of integration sites [21]. Nevertheless, mutations affecting the sequences located at the 3′ border of nucleosome 1, which increase its stability, produce a general reduction in basal transcription, irrespective of the integration site [57]. In contrast, the deletion of 60 nucleotides within the sequence covered by this nucleosome, which presumably destabilize it, makes basal transcription even more dependent on the integration site than the wild type [57]. This data is fully compatible with our yeast results and provides support for chromatin configuration playing a role in repressing HIV-1 transcription at the level of early elongation. A similar mechanism has been reported for the human *hsp70* gene [58], where the repressive chromatin configuration covering its transcribed region becomes remodeled during activation, allowing poised RNApolII to complete elongation [59].

The most relevant conclusion of our genetic analysis is the involvement of chromatin-reassembly factors in repressing HIV-1 basal transcription. We have confirmed that their function is not restricted to our chimeric yeast-HIV system but they are also playing a role in HIV-1 basal transcription in human cells. It has been reported that integration into regions of compacted chromatin, i.e. centromeric heterochromatin, causes HIV promoter inactivity, probably due to the inaccessibility of the basal transcriptional machinery or the inability of transcription factors to overcome the repressive chromatin state imposed [42]. In the latent integrations, this repressive state can be overcome by exogenous stimulation with mitogens or cytokines. We have found that, by knocking-down Spt6 and Chd1, the HIV promoter integrated in the context of latency increases its level of expression.

When we deplete Spt6 or Chd1 from the heterogeneous population of HIV latently infected cells, only a small proportion of integrant promoters are activated. This indicates that only a subset of integrations are either negatively regulated by these factors, or able to be reactivated by solely depleting them. In some chromatin environments, transcription may be completely inhibited, such that chromatin disruption by basal transcription would not be an issue and chromatin reassembly factors would play no role. Similarly, TSA treatment and YY1 depletion are not able to reactivate all latent integrations. We had predicted that individual integrations would have a more consistent response to the depletion of reassembly factors. In fact, we observed that individual clones responded differently, both amongst themselves and to the two distinct shRNAs. Still, not all cells of a clonal population respond equally. This behavior is also observed in response to TSA and resembles position effect variegation [42].

Depletion of Spt6 and Chd1 may cause a deficit of chromatin reassembly in those rare events, during non-activated conditions, in which RNApolII would be able to overcome the chromatin barrier (Figure 5A). This chromatin change would facilitate additional rounds of transcription under non-activating conditions and, eventually, an increase in transcription factor loading and PIC assembly. If transcription were enough to produce some Tat protein, then promoter activity would be reinforced. Inhibition of HDAC activity with TSA has a similar effect, supporting the idea that discrete modifications of chromatin compactness may be sufficient to switch a repressed provirus to active.

It has been shown absence of chromatin reassembly factors in yeast provokes activation of cryptic promoters located in the body of transcribed genes. In the absence of transcription, no PIC assembles on these cryptic promoters, due to the inhibitory action of chromatin; under active transcription, chromatin reassembly factors ensure rapid nucleosome deposition after RNApol II passage, avoiding the activation of cryptic promoters [54],[60]. HIV proviruses integrated in highly transcribed genes are usually latent [61]. Several mechanisms of transcriptional interference have been proposed to explain this phenomenon [62]. It has been shown that read-through transcription from an upstream promoter can interfere with HIV transcription by disturbing PIC assembly [63]. Recent published evidence indicates that transcriptional interference is caused by the elongating form of RNA pol II, transcribing through the latent HIV copy [64],[65]. This situation parallels that of yeast cryptic promoter and, accordingly, it is likely that chromatin reassembly factors also play an important role in maintaining HIV latency by transcriptional interference (Figure 5B). The integration site of one of the individual clones tested in our depletion experiments (clon 27) is a highly transcribed gene. We found a clear reactivation of the latent HIV copy of this clone when chromatin reassembly factors were depleted. In fact, when Spt6 was depleted, this clone showed the maximal reactivation level reached in the whole set of experiments.

When taken together, our results indicate that Spt6 and Chd1 participate in the mechanism that controls the equilibrium between activation and repression of HIV-1 expression when the provirus is integrated in the human genome, which depends greatly on the chromatin environment of the integration site. Disturbance of this equilibrium, by depleting chromatin reassembly factors for example, makes some of the latent integrations become activated. These factors may also play a general role in repressing basal transcription throughout the human genome. Therefore, the specificity of chromatin reassembly factors in repressing viral basal transcription should be carefully evaluated before considering them as therapeutic targets against HIV-1 latency.

## Materials and Methods

### Yeast Strains, Plasmids, and Media

Yeast strains used are listed in Table S1 and are isogenic to the S288C derivative BY4741 [66]. Plasmids are described in Table S2. Yeast cells were grown following standard procedures [67].

### Measurement of mRNA Levels

mRNA levels were measured by Northern analysis as described [29].

### Western Blots

Laemmli boiled crude extracts were run on a 10% SDS-polyacrylamide gel and transferred to nylon membranes (Hybond-ECL). After blocking with Tris-buffered saline containing 0.1% Tween 20 and 5% milk, proteins were detected using anti-FLAG antibodies (monoclonal, Santa Cruz) and human cyclin T1 (polyclonal, Santa Cruz) and peroxidase-conjugated goat anti-mouse and rabbit anti-goat IgG respectively. Blots were washed with tris-buffered saline and 0.1% Tween 20, and developed by enhanced chemiluminescence reactions (PIERCE). Signals were detected with Hyperfilms ECL (Amersham), exposing from 15 sec to 5 min.

### Chromatin Immunoprecipitation Assays

ChIP analyses of Rpb1-Myc were performed using a monoclonal anti-cMyc antibody (9E10) as described previously [55]. For crosslinking, cells were treated with 1% formaldehyde for 15 min at room temperature. As a non-transcribed control, we amplified a region adjacent to *FUS1*. Primer mixes were empirically adjusted for balanced signals. Immunoprecipitation was defined as the ratio of each gene-specific product in relation to that of the non-transcribed region, always after normalization with the signal of its corresponding whole-cell extract. Several dilutions of the whole cell extract were tested to ensure that the assays were in the linear range.

Spt16-Myc, Spt6-HA and Chd1-HA abundances on Ty1-HIV were assayed as indicated, by using ChIP with monoclonal anti-cMyc (9E10) or anti-HA antibodies and protein A-Sepharose. We used 20- to 30-bp oligonucleotides for PCR amplification of fragments of the Ty1 promoter (positions −157 to −107, relative to the transcription start site) and the 5′HIV-TR (positions +102 to +162). Real-time quantitative PCR was performed with SYBR green dye in the 7500 Real Time PCR system of Applied Biosystems by following the manufacturer's instructions. A non-transcribed telomeric fragment was used to normalize the signals. No-antibody controls were performed to exclude unspecific amplification.

### Chromatin Analysis

Yeast spheroplasts and micrococcal nuclease digestions were performed according to [68] with the modifications of [69]. Spheroplasts were prepared from mid-log phase cultures grown in SC-Ura with 2% glucose. Cells were lysed and immediately digested with 7.5 to 125 mU of micrococcal nuclease. Digested DNA was resolved in 1.5% agarose gels and analyzed by Southern blot with the probes indicated in Figure S2. Alternatively, DNA was quantified by real time PCR performed with SYBR green dye in a Applied Biosystems 7500 Real Time PCR system, following the manufacturer's instructions. In order to correct for the sequence-specificity of MNase, naked-DNA samples digested with MNase were also quantified by real-time PCR. The chromatin/naked-DNA ratio was considered to be a valid estimation of chromatin-dependent resistance to MNase.

### shRNA-Mediated Depletion of hSpt6 and hChd1 and HIV Expression Analysis

Pools or particular clones of Jurkat cells latently infected with an HIV-derived minigenome [42] were infected with Control, YY1, Spt6 or Chd1 shRNA-expressing lentiviruses (vector pLKO.1-Puro) obtained from Sigma (MISSION™ shRNAs). The protocol for viral particles production and cell infections has been described elsewhere [42]. Upon puromycin (2 mg/ml) selection of infected cells, HIV reactivation was followed by FACS analysis of GFP-positive cells. shRNA-mediated inhibition was tested by Western blot with specific anti-human YY1 antibodies (Santa Cruz sc-281), Spt6 (Abcam ab32820) and Chd1 (Abnova H00001105-A01). Anti-tubulin antibody was from Sigma.

A pool of latently infected HeLa cells was constructed as previously reported [42] and infected with several Spt6 and Chd1 shRNA-expressing vectors. Further details are described in the legend of the Figure S6.

## Supporting Information

Figure S1 Inhibitory effect of the 5′HIV-TR on basal transcription in three different yeast genetic backgrounds. mRNA samples from three different wild-type yeast strains, transformed with plasmids pTy1-HIV and pTy1-HIVTARless, were resolved in agarose gels and analyzed by Northern blotting. Quantification of the signals is shown, after normalizing with the levels of 25S rRNA.
(2.90 MB TIF)

Figure S2 The 5′HIV-TR is protected against MNase digestion in yeast. Spheroplasts of BY4741 cells containing pTy1-HIV and pTy1-HIVTARless were lysed and digested with increasing concentrations of MNase. After purification, DNA was resolved in agarose gels and hybridized with probes corresponding to the positions indicated in the diagram. The arrow points to the detected DNA fragments of subnucleosomal size.
(3.51 MB TIF)

Figure S3 Induction of Ty1 expression by 6-azauracil. Yeast cells of BY4741 and an isogenic strain lacking TFIIS (*dst1*Δ), transformed with the URA3-containing plasmid pRS416, were grown to mid-log phase in minimal medium without uracil. Samples were taken at the indicated times, after adding 6-azauracil (100 µg/ml). mRNA was extracted and analyzed by Northern blot with a Ty1 probe. Signals were normalized in accordance with the amounts of 25S rRNA. Averages of three independent experiments are shown.
(2.31 MB TIF)

Figure S4Effect of P-TEFb on GAl1-HIV expression in the presence of Tat. (A) Expression of human cyclin T1 and human FLAG-CDK9 in yeast was verified by Western blot with anti-hCycT1 an anti-FLAG antibodies. (B) Expression of functional Tat in yeast was verified following a triple-hybrid strategy, according to Fraldi et al (J Cell Biochem 36: 247–253). Expression of *lac* 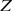 was detected when Tat was expressed in the MVcoat-b yeast strain. (C) W303-1A cells transformed with plasmids pGAL1-HIV, alone or together with combinations of p415GPD-CycT1, p414GPD-Cdk9 and p413GPD-Tat, were grown to mid-log phase in minimal medium with glycerol and lactate as carbon sources, and incubated for 90 min in the presence of 2% galactose. mRNA samples were taken at different times and analyzed by Northern blot with an HIV probe. Signals were normalized in accordance with the amounts of 25S rRNA. Averages of four independent experiments are shown.(14.57 MB TIF)

Figure S5 The inhibitory role of the 5′HIV-TR is compromised in mutants affecting co-transcriptional chromatin reassembly. mRNA samples of the indicated mutants, transformed with pTy1-HIV and pTy1-HIVTARless, were analyzed by Northern blot, as in Figure 1A. Averages of at least three different experiments are shown.
(3.28 MB TIF)

Figure S6 shRNA-mediated depletion of chromatin reassembly factors hSpt6 and hChd1 in a HeLa model of viral latency derepress the integrated HIV promoter. (A) Inducible HIV promoter in HeLa cells. HeLa cells were infected with a HIV minigenome, LTR-Tat-IRES-GFP-LTR. GFP-positive and GFP-negative cells were purified by cell sorting. Thereafter, GFP-negative cells were treated with TNFa overnight and resulting GFP-positive cells were purified to establish a population of latently infected cells. After several weeks in culture in the absence of TNFa, the majority of cells became GFP-negative. The response of this population to overnight treatment with PMA (10 nM), TSA (400 nM), TNFa (10 ng/ml), or a combination of the three is shown as percentage of cells that became GFP-positive, in comparison to untreated cells. (B) shRNA-mediated depletion of Spt6 activates the HIV promoter. HIV latently infected HeLa cells were infected with Control or Spt6 (#1, target sequence CGCCTTGTACTGTGAATTTAT)-shRNA expression lentivirus (pLKO.1-Puro, MISSION, Sigma). Upon puromycin (2 mg/ml) selection, depletion of this factor was tested in Western blot with specific antibodies and tubulin as a loading control (upper panel) and HIV reactivation was followed by FACS analysis of GFP-positive cells (lower panel). Spt6 depletion and HIV reactivation was observed at day 9 after infection, but was lost at day 19. (C) HIV reactivation is achieved with several different shRNAs against Spt6. As in (B), lentiviruses for the expression of two additional Spt6 shRNAs (#2, CCCTTGAAGAAATCTTGGAAA and #3, GCCCACCTTCATCCCTTATTT) were integrated into latently infected HeLa cells at two different multiplicities of infection (here called *low* and *high*, *high* being 9-fold higher than *low*). Analysis of Spt6 depletion by Western blot and of the percentage of HIV-reactivated cells was performed 9 days after infection. (D) shRNA-mediated depletion of Chd1 activates the HIV promoter. HIV latently infected HeLa cells were infected with Control or Chd1 (#1, GCGGTTTATCAAGAGCTATAA; #2, CCACTCTTACTTCCTGGCAAA; #3, GCAGTTGTGATGAAACAGAAT and #4, CCATCGTGATTGGGATCACTA)-shRNA expression lentiviruses (pLKO.1-Puro, MISSION, Sigma), at two different multiplicities of infection. Upon puromycin (2 mg/ml) selection, the repressive effect of these shRNAs was tested by RT-quantitative PCR with gene-specific oligonucleotides and GAPDH as a control (upper panel) and HIV reactivation was followed by FACS analysis of GFP-positive cells (lower panel). The two shRNAs with a stronger capability of knocking-down Chd1 promoted clearer reactivation of latent HIV.
(26.14 MB TIF)

Table S1 Yeast strains.
(0.12 MB DOC)

Table S2 Plasmids used in this work.
(0.13 MB DOC)

Table S3 Primers for real-time PCR.
(0.11 MB DOC)
