# Correction: Yeast Genetic Analysis Reveals the Involvement of Chromatin Reassembly Factors in Repressing HIV-1 Basal Transcription

**DOI:** 10.1371/annotation/45587774-5d7c-4023-97b0-96833be7ad9a

**Published:** 2009-01-26

**Authors:** Manuela Vanti, Edurne Gallastegui, Iñaki Respaldiza, Alfonso Rodríguez-Gil, Fernando Gómez-Herreros, Silvia Jimeno-González, Albert Jordan, Sebastián Chávez

The following information was omitted from the Funding section: This work was also supported by a grant from the 'Fundacion para la investigacion y prevencion del SIDA en España' (FIPSE) to AJ. The current address for Edurne Gallastegui and Albert Jordan was not indicated. They are both at: Institut de Biologia Molecular de Barcelona (IBMB-CSIC), Barcelona, Spain.

