# Supplementary figures and images for: Yeast Genetic Analysis Reveals the Involvement of Chromatin Reassembly Factors in Repressing HIV-1 Basal Transcription
Source: PLoS Genet. 2009 Jan 16;5(1):e1000339. doi: 10.1371/journal.pgen.1000339 (PMC2613532; doi:10.1371/journal.pgen.1000339)

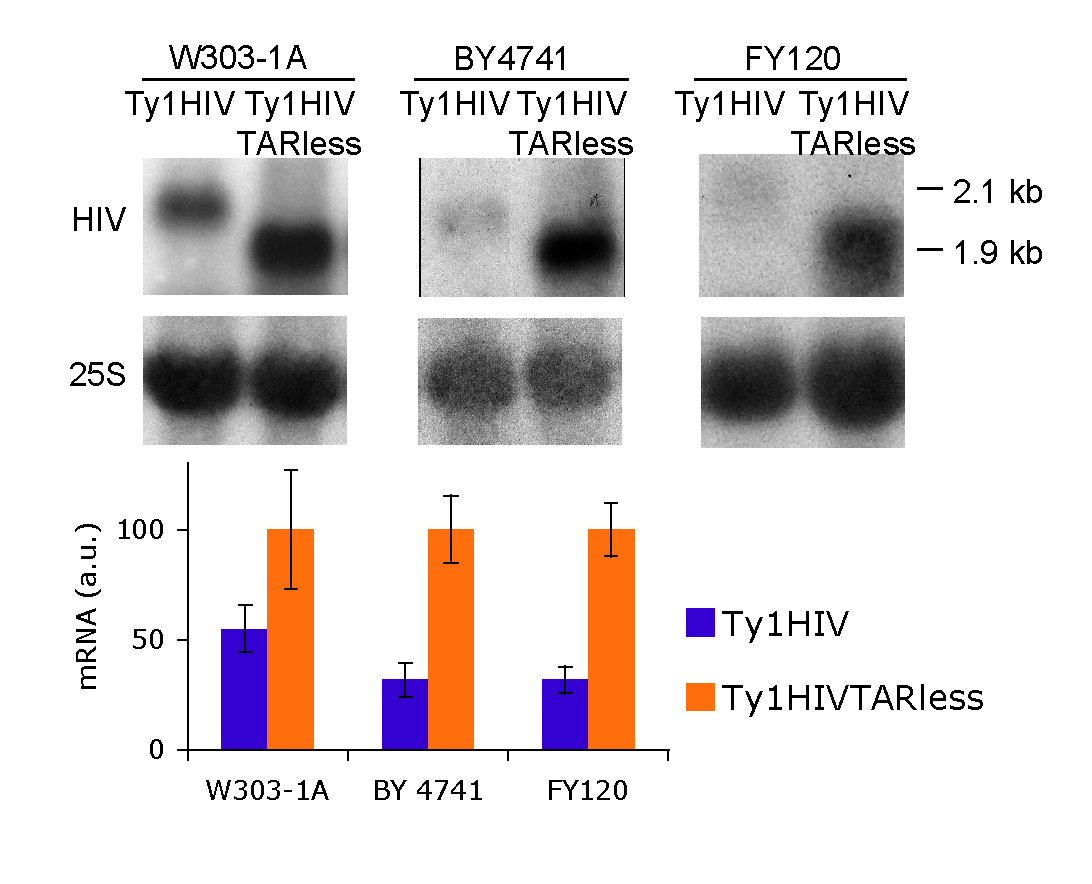

Supplement: Figure S1 — Inhibitory effect of the 5′HIV-TR on basal transcription in three different yeast genetic backgrounds. mRNA samples from three different wild-type yeast strains, transformed with plasmids pTy1-HIV and pTy1-HIVTARless, were resolved in agarose gels and analyzed by Northern blotting. Quantification of the signals is shown, after normalizing with the levels of 25S rRNA. (2.90 MB TIF) [file pgen.1000339.s001.tif]

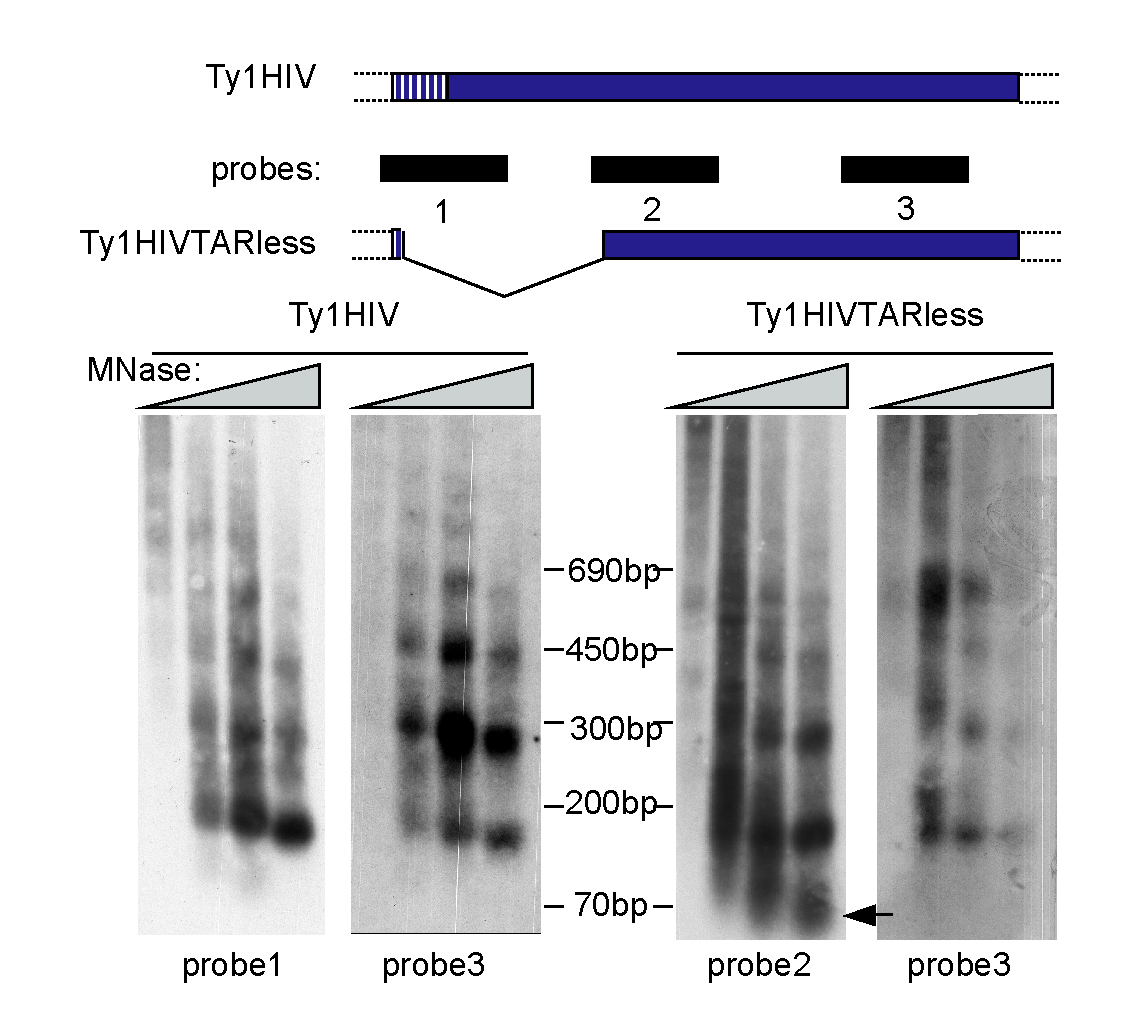

Supplement: Figure S2 — The 5′HIV-TR is protected against MNase digestion in yeast. Spheroplasts of BY4741 cells containing pTy1-HIV and pTy1-HIVTARless were lysed and digested with increasing concentrations of MNase. After purification, DNA was resolved in agarose gels and hybridized with probes corresponding to the positions indicated in the diagram. The arrow points to the detected DNA fragments of subnucleosomal size. (3.51 MB TIF) [file pgen.1000339.s002.tif]

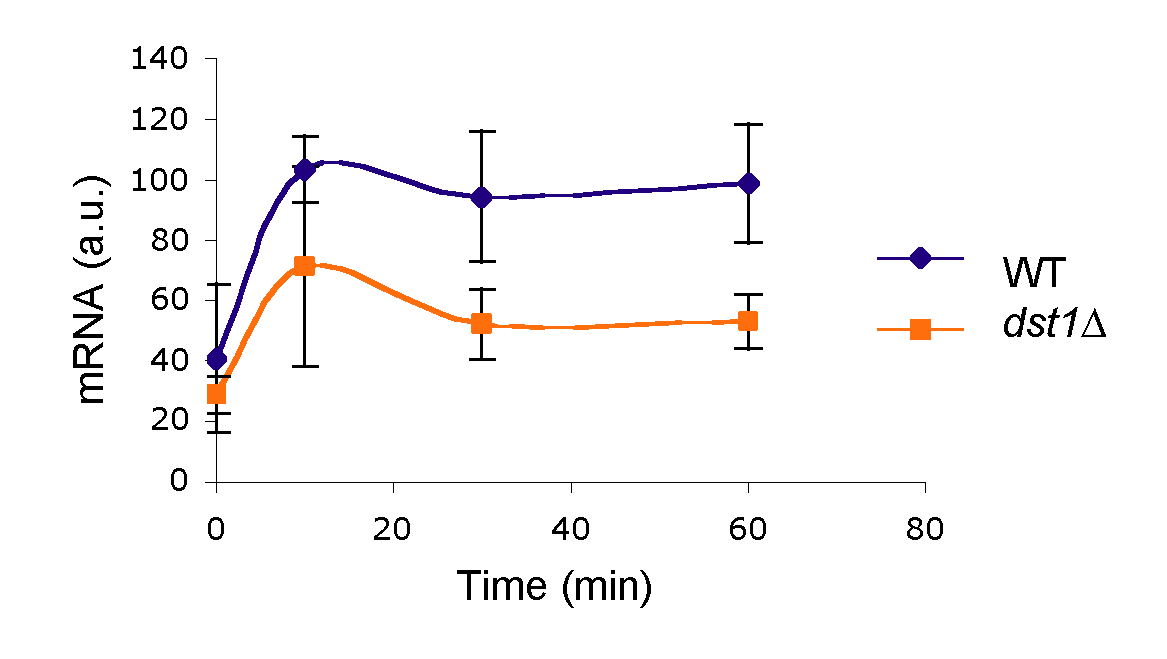

Supplement: Figure S3 — Induction of Ty1 expression by 6-azauracil. Yeast cells of BY4741 and an isogenic strain lacking TFIIS (dst1Δ), transformed with the URA3-containing plasmid pRS416, were grown to mid-log phase in minimal medium without uracil. Samples were taken at the indicated times, after adding 6-azauracil (100 µg/ml). mRNA was extracted and analyzed by Northern blot with a Ty1 probe. Signals were normalized in accordance with the amounts of 25S rRNA. Averages of three independent experiments are shown. (2.31 MB TIF) [file pgen.1000339.s003.tif]

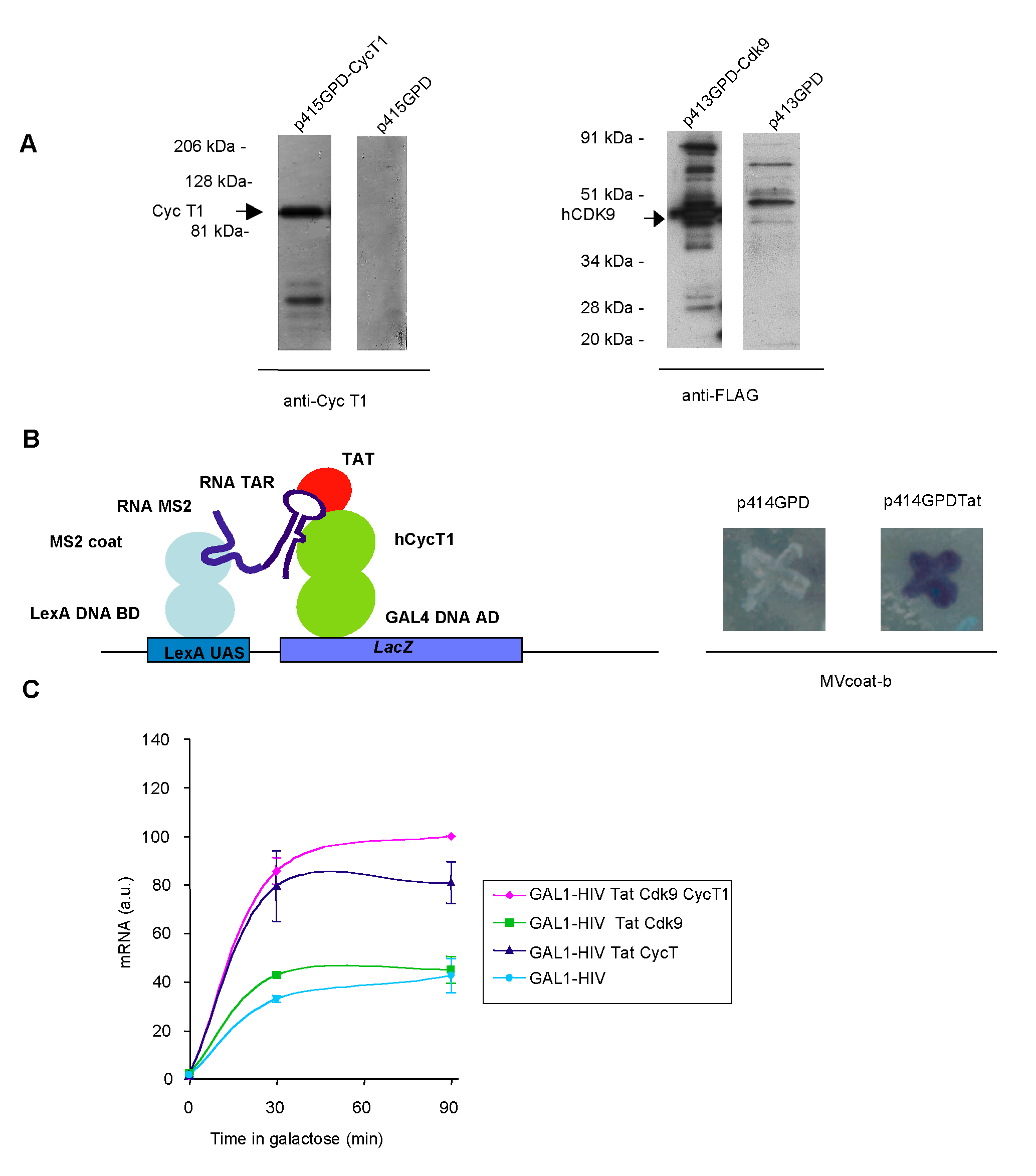

Supplement: Figure S4 — Effect of P-TEFb on GAl1-HIV expression in the presence of Tat. (A) Expression of human cyclin T1 and human FLAG-CDK9 in yeast was verified by Western blot with anti-hCycT1 an anti-FLAG antibodies. (B) Expression of functional Tat in yeast was verified following a triple-hybrid strategy, according to Fraldi et al (J Cell Biochem 36: 247–253). Expression of lac was detected when Tat was expressed in the MVcoat-b yeast strain. (C) W303-1A cells transformed with plasmids pGAL1-HIV, alone or together with combinations of p415GPD-CycT1, p414GPD-Cdk9 and p413GPD-Tat, were grown to mid-log phase in minimal medium with glycerol and lactate as carbon sources, and incubated for 90 min in the presence of 2% galactose. mRNA samples were taken at different times and analyzed by Northern blot with an HIV probe. Signals were normalized in accordance with the amounts of 25S rRNA. Averages of four independent experiments are shown. (14.57 MB TIF) [file pgen.1000339.s004.tif]

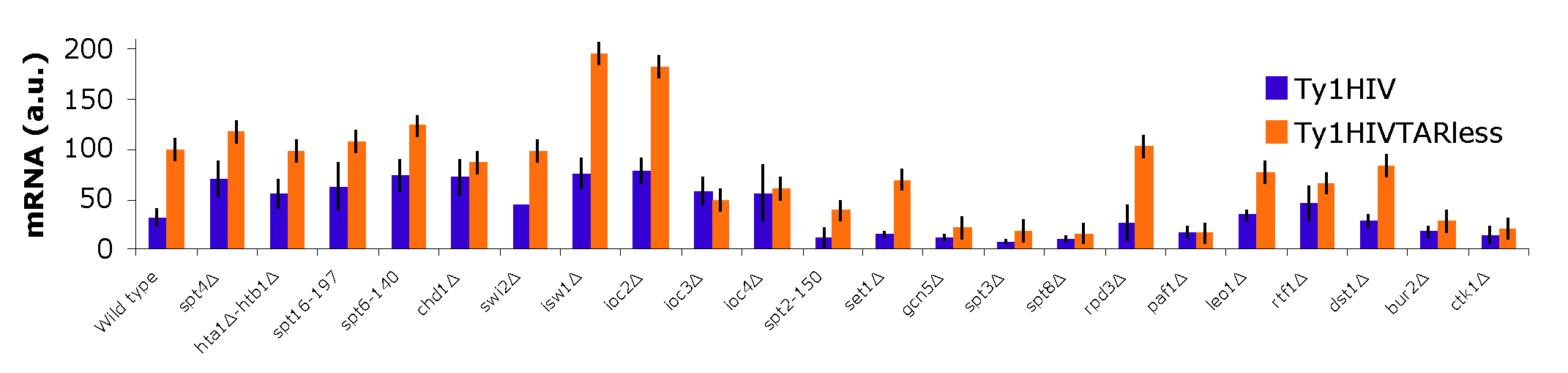

Supplement: Figure S5 — The inhibitory role of the 5′HIV-TR is compromised in mutants affecting co-transcriptional chromatin reassembly. mRNA samples of the indicated mutants, transformed with pTy1-HIV and pTy1-HIVTARless, were analyzed by Northern blot, as in Figure 1A. Averages of at least three different experiments are shown. (3.28 MB TIF) [file pgen.1000339.s005.tif]

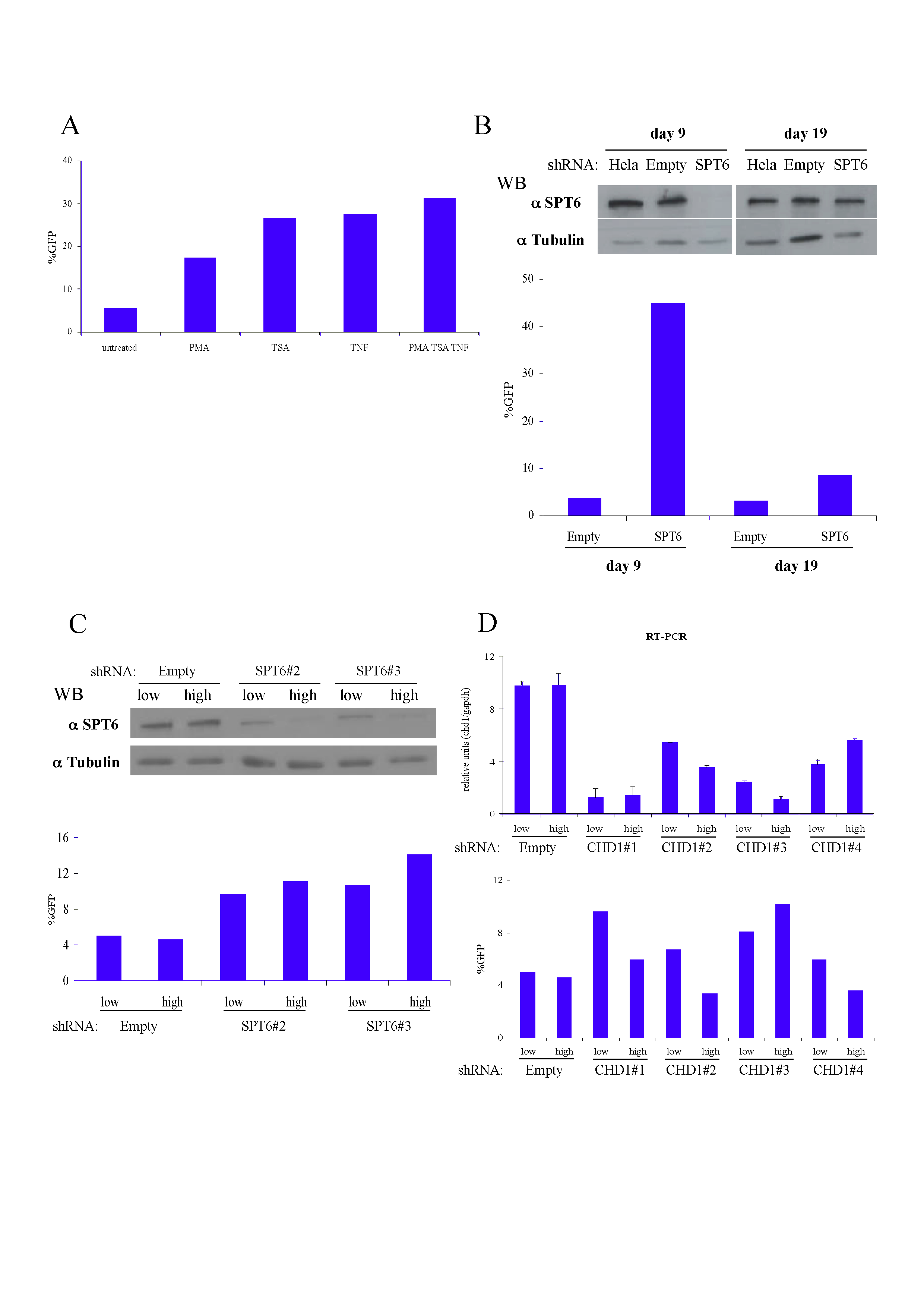

Supplement: Figure S6 — shRNA-mediated depletion of chromatin reassembly factors hSpt6 and hChd1 in a HeLa model of viral latency derepress the integrated HIV promoter. (A) Inducible HIV promoter in HeLa cells. HeLa cells were infected with a HIV minigenome, LTR-Tat-IRES-GFP-LTR. GFP-positive and GFP-negative cells were purified by cell sorting. Thereafter, GFP-negative cells were treated with TNFa overnight and resulting GFP-positive cells were purified to establish a population of latently infected cells. After several weeks in culture in the absence of TNFa, the majority of cells became GFP-negative. The response of this population to overnight treatment with PMA (10 nM), TSA (400 nM), TNFa (10 ng/ml), or a combination of the three is shown as percentage of cells that became GFP-positive, in comparison to untreated cells. (B) shRNA-mediated depletion of Spt6 activates the HIV promoter. HIV latently infected HeLa cells were infected with Control or Spt6 (#1, target sequence CGCCTTGTACTGTGAATTTAT)-shRNA expression lentivirus (pLKO.1-Puro, MISSION, Sigma). Upon puromycin (2 mg/ml) selection, depletion of this factor was tested in Western blot with specific antibodies and tubulin as a loading control (upper panel) and HIV reactivation was followed by FACS analysis of GFP-positive cells (lower panel). Spt6 depletion and HIV reactivation was observed at day 9 after infection, but was lost at day 19. (C) HIV reactivation is achieved with several different shRNAs against Spt6. As in (B), lentiviruses for the expression of two additional Spt6 shRNAs (#2, CCCTTGAAGAAATCTTGGAAA and #3, GCCCACCTTCATCCCTTATTT) were integrated into latently infected HeLa cells at two different multiplicities of infection (here called low and high, high being 9-fold higher than low). Analysis of Spt6 depletion by Western blot and of the percentage of HIV-reactivated cells was performed 9 days after infection. (D) shRNA-mediated depletion of Chd1 activates the HIV promoter. HIV latently infected HeLa cells were infecte [file pgen.1000339.s006.tif]
