# Supplementary material for: Yeast Genetic Analysis Reveals the Involvement of Chromatin Reassembly Factors in Repressing HIV-1 Basal Transcription
Source: PLoS Genet. 2009 Jan 16;5(1):e1000339. doi: 10.1371/journal.pgen.1000339 (PMC2613532; doi:10.1371/journal.pgen.1000339)
Supplement: Table S1 — Yeast strains. (0.12 MB DOC) [file pgen.1000339.s007.doc]

**Table S1. Yeast strains.**

| Strain | Relevant genotype | Reference |
| --- | --- | --- |
| W303-1A | *MATa ade2-1 can1-100 his3-11,15 leu2-3,112 trp1-1 ura3-1* | R. Rothstein |
| BY4741 | *MATa his3Δ1, leu2Δ0, met15∆0, ura3Δ0* | [1] |
| FY120 | *MATa ura3, leu2Δ1, his4-912δ, lys2-128δ* | [2] |
| FY137 | *MATa ura3, his4-912δ, lys2-128δ spt6-140* | [2] |
| FY348 | *MATa ura3, leu2Δ1, his4-912δ, lys2-128δ spt16-197* | [3] |
| FY710 | *MATa ura3, leu2Δ1, his4-912δ, lys2-128δ hta1-htb1::LEU1* | [4] |
| FY232 | *MATa ura3, ade2, his4-912δ, lys2-128δ spt2-150* | [5] |
| L-40COAT III | *MATa ade2-1, his3Δ200, leu2-3,112, trp1-Δ1, LYS2::(lexAop)_4_-HIS3, ura3::(lexAop)_8_-lacZ, lexA-MS2coat (TRP1)* | [6] |
| MVcoat-b | *MATa, his3Δ200, leu2-3,112, trp1-Δ1, lys2∆0, ura3::(lexAop)_8_-lacZ, lexA-MS2coat (TRP1)* | This work |
| ARG3 | BY4741 *YDR007::kanMX (trp1Δ) RPB1myc**18::klTRP1* | This work |
| SJY25 | BY4741 *trp1::kanMX4 SPT16-myc18::klTRP1* | [7] |
| DBY871 | W303-1A *SPT6::HA* | D. Bentley |
| DBY969 | BY4741 *chd1∆* pRS316*CHD1::HA* | D. Bentley |

Other yeast strains, not listed in Table S1, are derivative of BY4741 and were obtained from EUROSCARF (<http://web.uni-frankfurt.de/fb15/mikro/euroscarf/col_index.html>).

**References**

1. Brachmann CB, Davies A, Cost GJ, Caputo E, Li J, et al. (1998) Designer deletion strains derived from Saccharomyces cerevisiae S288C: a useful set of strains and plasmids for PCR-mediated gene disruption and other applications. Yeast 14: 115-132.

2. Hartzog GA, Wada T, Handa H, Winston F (1998) Evidence that Spt4, Spt5, and Spt6 control transcription elongation by RNA polymerase II in Saccharomyces cerevisiae. Genes Dev 12: 357-369.

3. Malone EA, Clark CD, Chiang A, Winston F (1991) Mutations in SPT16/CDC68 suppress cis- and trans-acting mutations that affect promoter function in Saccharomyces cerevisiae. Mol Cell Biol 11: 5710-5717.

4. Clark-Adams CD, Norris D, Osley MA, Fassler JS, Winston F (1988) Changes in histone gene dosage alter transcription in yeast. Genes Dev 2: 150-159.

5. Winston F, Chaleff DT, Valent B, Fink GR (1984) Mutations affecting Ty-mediated expression of the HIS4 gene of Saccharomyces cerevisiae. Genetics 107: 179-197.

6. Fraldi A, Licciardo P, Majello B, Giordano A, Lania L (2001) Distinct regions of cyclinT1 are required for binding to CDK9 and for recruitment to the HIV-1 Tat/TAR complex. J Cell Biochem 36: 247-253.

7. Jimeno-Gonzalez S, Gomez-Herreros F, Alepuz PM, Chavez S (2006) Gene-specific requirement for FACT during transcription is related to the chromatin organization of the transcribed region. Mol Cell Biol.
