# Supplementary material for: Yeast Genetic Analysis Reveals the Involvement of Chromatin Reassembly Factors in Repressing HIV-1 Basal Transcription
Source: PLoS Genet. 2009 Jan 16;5(1):e1000339. doi: 10.1371/journal.pgen.1000339 (PMC2613532; doi:10.1371/journal.pgen.1000339)
Supplement: Table S2 — Plasmids used in this work. (0.13 MB DOC) [file pgen.1000339.s008.doc]

Table S2. Plasmids used in this work.

| Plasmid | Relevant features | Reference |
| --- | --- | --- |
| p413GPD | CEN, *HIS3*, GPD promoter. | [1] |
| p414GPD | CEN, *TRP1*, GPD promoter. | [1] |
| p415GPD | CEN, *LEU2*, GPD promoter. | [1] |
| p416GAL1 | CEN, *URA3*, GAL1 promoter. | [1] |
| pFLAG-CMV2-CDK9 | FLAG-hCDK9 | K. Jones laboratory |
| pSV-Tat | Tat cDNA | [2] |
| pCMV-CycT1 | Expression plasmid for hCycT1 | K. Jones laboratory |
| pNL43 | HIV-1 NL43 full genome | F. Luque laboratory |
| p413GPD-CDK9-FLAG | Yeast expression plasmid for FLAG-hCDK9, derived from p413GPD | This work |
| p414GPD-Tat | Yeast expression plasmid for Tat, derived from p414GPD | This work |
| p415GPD-CycT1 | Yeast expression plasmid for hCycT1, derived from p415GPD | This work |
| pGAL1-HIV | 5’ end of the HIV-1 transcribed region (nucleotides 433-1145 of HIV-1 NL43) fused to the coding region of yeast *PHO5* and driven by the GAL1 promoter. Derived from p416GAL1. | This work |
| pGAL1-HIVTARless | Deleted version of pGAL1-HIV containing a 531-1145 fragment of the NL43 HIV-1 genome. | This work |
| pTy1-HIV | 5’ end of the HIV-1 transcribed region (nucleotides 454-1145 of HIV-1 NL43) fused to the coding region of yeast *PHO5* and driven by the Ty1-4 promoter. The Ty1-4 promoter was amplified by PCR from W303-1A (nucleotides 1097137-1097350 of chromosome IV) and inserted in p416GAL1, replacing the *GAL1* promoter. | This work |
| pTy1-HIVTARless | Deleted version of pTy1-HIV, lacking the 474-678 fragment of the NL43 HIV-1 genome. | This work |
| pACTII-CycT1 | Yeast expression plasmid for a fusion between hCycT1 and the activation domain of Gal4, driven by the constitutive ADH1 promoter. | [3] |
| pIIIA-TAR-MS2 | Yeast expression plasmid for a fusion between TAR and the MS2 RNA | [3] |

**References**

1. Mumberg D, Muller R, Funk M (1995) Yeast vectors for the controlled expression of heterologous proteins in different genetic backgrounds. Gene 156: 119-122.

2. Kao SY, Calman AF, Luciw PA, Peterlin BM (1987) Anti-termination of transcription within the long terminal repeat of HIV-1 by tat gene product. Nature 330: 489-493.

3. Fraldi A, Licciardo P, Majello B, Giordano A, Lania L (2001) Distinct regions of cyclinT1 are required for binding to CDK9 and for recruitment to the HIV-1 Tat/TAR complex. J Cell Biochem 36: 247-253.
