# Supplementary material for: Yeast Genetic Analysis Reveals the Involvement of Chromatin Reassembly Factors in Repressing HIV-1 Basal Transcription
Source: PLoS Genet. 2009 Jan 16;5(1):e1000339. doi: 10.1371/journal.pgen.1000339 (PMC2613532; doi:10.1371/journal.pgen.1000339)
Supplement: Table S3 — Primers for real-time PCR. (0.11 MB DOC) [file pgen.1000339.s009.doc]

Table S3. Primers for real-time PCR

| Forward | Position (1) | Reverse | Position (1) |
| --- | --- | --- | --- |
| tctctggctaactagggaaccca | 38 | gcactcaaggcaagctttattga | 94 |
| aagcctcaataaagcttgccttg | 67 | agtcacacaacagacgggcac | 127 |
| agtgtgtgcccgtctgttgtg | 102 | tgactaaaagggtctgagggatctcta | 162 |
| gagatccctcagacccttttagtca | 138 | ttcaagtccctgttcgggc | 205 |
| gtggcgcccgaacagg | 182 | ttgccgtgcgcgctt | 270 |
| caggactcggcttgctgaag | 239 | caccagtcgccgccc | 293 |
| cgactggtgagtacgccaaaa | 285 | atctctctccttctagcctccgc | 338 |
| gcggaggctagaaggagagagat | 316 | taataccgacgctctcgcacc | 360 |
| gcgagagcgtcggtattaagc | 343 | tttccccctggccttaacc | 413 |
| aaaattcggttaaggccaggg | 388 | ggattaactgcgaatcgttctagct | 479 |
| gggagctagaacgattcgcag | 452 | ctgatgtctctaaaaggccaggatta | 499 |
| gattcgcagttaatcctggcct | 464 | ccagtatttgtctacagccttctgatgt | 520 |
| gagacatcagaaggctgtagacaaat | 490 | ggatggttgtagctgtcccag | 537 |
| actgggacagctacaaccatcc | 516 | aagttcttctgatcctgtctgaagg | 561 |
| acaaccatcccttcagacaggat | 528 | cctttgatgcacacaatcgaggact | 609 |
| cctctattgtgtgcatcaaaggatag | 588 | ggcttccttggtgtcttttacatcta | 636 |

(1) Position of the 5’ end of the oligo, relative to the +1 of the 5’HIV-TR
